# Improving Adherence and Clinical Outcomes in Self-Guided Internet Treatment for Anxiety and Depression: Randomised Controlled Trial

**DOI:** 10.1371/journal.pone.0062873

**Published:** 2013-07-03

**Authors:** Nickolai Titov, Blake F. Dear, Luke Johnston, Carolyn Lorian, Judy Zou, Bethany Wootton, Jay Spence, Peter M. McEvoy, Ronald M. Rapee

**Affiliations:** 1 Centre for Emotional Health, Department of Psychology, Macquarie University, Sydney, Australia; 2 Specialist Clinical Psychologist, Centre for Clinical Interventions and School of Psychology, University of Western Australia, Perth, Australia; Linkoping University, Sweden

## Abstract

**Background:**

Depression and anxiety are common, disabling and chronic. Self-guided internet-delivered treatments are popular, but few people complete them. New strategies are required to realise their potential.

**Aims:**

To evaluate the effect of automated emails on the effectiveness, safety, and acceptability of a new automated transdiagnostic self-guided internet-delivered treatment, the Wellbeing Course, for people with depression and anxiety.

**Method:**

A randomised controlled trial was conducted through the website: www.ecentreclinic.org. Two hundred and fifty seven people with elevated symptoms were randomly allocated to the 8 week course either with or without automated emails, or to a waitlist control group. Primary outcome measures were the Patient Health Questionnaire 9-Item (PHQ-9) and the Generalized Anxiety Disorder 7-Item (GAD-7).

**Results:**

Participants in the treatment groups had lower PHQ-9 and GAD-7 scores at post-treatment than controls. Automated emails increased rates of course completion (58% vs. 35%), and improved outcomes in a subsample with elevated symptoms.

**Conclusions:**

The new self-guided course was beneficial, and automated emails facilitated outcomes. Further attention to strategies that facilitate adherence, learning, and safety will help realise the potential of self-guided interventions.

**Trial Registration:**

Australian and New Zealand Clinical Trials Registry ACTRN12610001058066

## Introduction

The World Mental Health Surveys indicate that globally more than 500 million people each year meet diagnostic criteria for anxiety and depressive disorders [1]. Effective psychological treatments for these conditions are increasingly available via the internet (internet psychotherapy; iPT), which offers improved accessibility and convenience for both patients and clinicians [2].

Disorder-specific iPT interventions are clinically effective for adults with anxiety disorders and depression when guided by a therapist [3], [4], [5], [6]. Recent work indicates that transdiagnostic iPT interventions, which treat symptoms of both anxiety and depression in the same intervention, are also efficacious when administered by a therapist [7], [8], [9]. However, many consumers prefer and opt for self-guided iPT over therapist-guided versions. For example, recent research by a large iPT clinic found that only 75 of 2660 (i.e., 2%) consecutive participants elected for therapist-guided iPT over self-guided iPT [10].

Publicly available self-guided iPT interventions are popular. For example, the Panic Center (www.paniccentre.net) and Moodgym (www.moodgym.anu.edu.au), which offer disorder-specific and self-guided online interventions, each have more than 400,000 registered users. Unfortunately, while data indicate that those who adhere and complete these interventions obtain good outcomes [11], [12], [13], the full public health potential of self-guided iPT has arguably not yet been realised. Typically more than 90% of consumers withdraw after two sessions and, thus, are unlikely to receive a ‘dose’ of therapeutic content sufficient to lead to sustained improvement. Self-guided transdiagnostic iPT interventions which include strategies that facilitate adherence have considerable potential for greater outcomes while providing clinically and cost effective treatment to large numbers of people.

One strategy for improving adherence is to utilise reminders that promote both exposure to therapeutic content and practice of therapeutic skills [14]. Surprisingly, very few self-guided iPT interventions provide reminders. However, a trial of self-guided iPT for depression [15] demonstrated that reminders increased clinical symptom reductions relative to an earlier trial without reminders [16] and evidence that effect was more pronounced in participants who were more severely depressed at pre-treatment. Similarly, comparisons across trials of iPT for adults with social phobia show that automated reminder emails increased completion rates and effect sizes relative to earlier trials without automated reminders [17], [18], [19]. Automated emails may be particularly suitable as a medium for providing reminders given the extremely low cost of emails and their ubiquity. Moreover, emerging studies are also indicating that emails can be used as a stand-alone self-help measure with sub-threshold depression to produce small but significant changes in symptom levels [20]. However, despite this emerging evidence and their potential, no studies have directly or systematically explored the ability of automated reminders in self-guided iPT to improve adherence and clinical outcomes.

The primary aim of the current study was to determine whether automated emails improved adherence and clinical outcomes during a new transdiagnostic self-guided iPT intervention, the *Wellbeing Course*, designed to treat symptoms of depression and anxiety. A secondary aim of the current study was to provide preliminary data about participant safety and the acceptability of the intervention. The study design simulated an automated and publically available website, where consumers applied and completed the intervention with no contact with staff. It was hypothesized that (1) automated emails would enhance completion rates and reductions on clinical symptoms, and (2) automated emails would be particularly beneficial for participants with elevated symptoms. No hypotheses were developed with respect to the acceptability or safety of the Wellbeing Course.

## Methods

The protocol for this trial and supporting CONSORT checklist are available as supporting information; see Checklist S1 and Protocol S1.

### Ethics Statement and Trial Registration

The study was approved by the Human Research Ethics Committee (HREC) of the University of New South Wales (UNSW, Sydney, Australia) and ratified by the Macquarie University HREC (Sydney, Australia; HREC Reference: 5201100226). The original protocol included telephone interviews at recruitment and a fourth group who received weekly contact with a therapist. However, in order to focus on the benefits of providing automatic emails during self-guided treatment and to simulate an entirely automated treatment process, the interviews and this fourth group were omitted from the final design. This change was approved by the UNSW HREC. The trial is registered with the Australian and New Zealand Clinical Trials registry as ACTRN12610001058066 and complies with updated CONSORT recommendations [21].

### Design and Sample Size

Eligible applicants were randomly allocated to one of two treatment groups or to a waitlist-deferred treatment control group. Participants in the Treatment Plus Automated Email Group (TEG; n = 100) received access to the Wellbeing Course with automated emails. Participants in the Treatment Group (TG; n = 106) received access to the Wellbeing Course without automated emails. The Control Group (Control; n = 51) received access to the same treatment as the TEG group following post-treatment.

A weighted randomisation procedure was employed in which 40% of successful applicants were assigned to each treatment group and 20% were assigned to the control group. A priori power analysis indicated that 246 participants would be sufficient to detect a between-treatment group (Cohen’s *d*) effect size (ES) difference ≥0.35 between the two treatment groups and an ES difference ≥0.55 between the treatment and control groups with alpha at.05 (one-tailed) and power of.80). These ESs were deemed sufficient, based on similar studies [22], [23] but more were recruited to hedge against attrition.

A colleague in another country used the website www.random.org to create a list of 300 randomly generated numbers. These were numbers were sorted by size and the highest 20% and lowest 40% were marked to indicated they referred to the control group and the TG, respectively. The remainder were marked to indicate they belong to the TEG. The list was then returned to its original order and was used to automatically assign applicants to a condition.

### Participants and Recruitment

Participants were automatically recruited from visitors to a research website that evaluates internet-delivered treatments (www.ecentreclinic.org). After reading about the study and providing informed consent, applicants completed online questionnaires enquiring about symptoms of mood and anxiety, demographic details and treatment history.

During the seven weeks of recruitment in mid-2011, 381 individuals applied for this trial, and 274 met the following inclusion criteria: (i) Resident of Australia and at least 18 years of age; (ii) self-identified as having a principal complaint of depression, generalised anxiety disorder (GAD), social phobia, or panic disorder as indicated by total scores on at least one of the following measures: Patient Health Questionnaire-9 Item (PHQ-9) >9; [24], [25] Generalised Anxiety Disorder-7 Item (GAD-7) >7; [26], [27] MINI Social Phobia Inventory (MINI-SPIN) >5; [28] Autonomic Nervous System Questionnaire (ANSQ) >1, [29] indicating likely diagnoses of depression, GAD, social phobia, and panic disorder, respectively; (iii) not currently participating in cognitive behavior therapy (CBT); (iv) no change in medications in the period 1 month prior to this study; (v) not currently experiencing a psychotic mental illness or severe symptoms of depression (defined as a total score ≥23 or responding >2 to Question 9 (suicidal ideation) on the PHQ-9; (vi) had reliable Internet access; (vii) provided personal contact details.

### Measures

The GAD-7, PHQ-9, MINI-SPIN and ANSQ were employed in the present trial as preliminary measures ensure participants had clinical level symptoms and were eligible for the trial. These measures are all widely used and all how high levels of sensitivity (i.e., ≥.75) and specificity (i.e., ≥.75), except the ANSQ which shows high levels of sensitivity (range:.94 to 1.00) but low levels of specificity (Range:.25 to.59) [24]–[29].

The primary outcome measures were severity of symptoms of depression and anxiety, measured by the PHQ-9 and GAD-7, which are based on the DSM-IV diagnostic criteria for depression and GAD, respectively. The GAD-7 has good convergent validity with other anxiety scales [25], is sensitive to DSM-IV congruent GAD, social phobia, and panic disorder, with increasing scores indicating greater symptom severity [27]. The GAD-7 is increasingly used in research and in large scale dissemination studies as a generic measure of change in anxiety symptoms [30], [31]. A total score of 10 on the PHQ-9 has been identified as an important threshold for identifying DSM-IV congruent depression with increasing scores indicating greater symptom severity, while psychometric studies indicate the measure is sensitive to change [24], [25], [32].

Secondary outcome measures included treatment satisfaction and completion rates (i.e., percentage in each treatment group who read the five online lessons of the Wellbeing Course within the 8 weeks of the course). Additional measures included changes in health status and service utilisation, which will be reported elsewhere.

TEG and TG participants completed the PHQ-9 and GAD-7 at pre-treatment, prior to each of the five lessons of the Wellbeing Course, at post-treatment, and again at 3-month follow-up. The satisfaction measures were administered at post-treatment. Controls completed the PHQ-9 and GAD-7 at the pre-and post-treatment time points of the treatment groups, and then began treatment.

### Intervention

The Wellbeing Course is a new five lesson transdiagnostic online intervention based on models of cognitive behavioural and interpersonal therapies. The original Wellbeing Program developed by the researchers [8], [9] was not available when the researchers moved institutions and, consequently, a new intervention was developed. This new course is based on a pragmatic model of psychotherapeutic change that assumes that symptoms of anxiety and depression are the result of unhelpful habits of thought and actions, that is, maladaptive cognitions and behaviours. Such unhelpful habits often develop over months or years and may become an entrenched part of a person’s life. This model also assumes that interventions that are structured, systematic, and which require adherence and commitment over several months are more likely to facilitate sustained improvements than sporadic or unstructured therapy sessions, which may only result in short-term symptom relief.

Thus, the Wellbeing Course is a structured intervention that participants complete over an 8 week period. Participants are strongly encouraged to learn about and practice the psychological skills taught in the course, and to adopt these into their everyday lives. The course systematically teaches core psychological skills that aim to increase the frequency of cognitions and behaviours that promote emotional health, and reduce those that maintain distressing symptoms. Examples of the former include realistic thinking skills, planning, and problem solving skills, assertive communication, behavioural activation, and graded exposure. Examples of the latter include patterns of catastrophic and self-defeating thinking, passive or aggressive communication styles, avoidance, and behavioural inhibition. By doing so, this Course targets symptoms common to anxiety disorders and depression. The Wellbeing Course was designed as a low intensity intervention, which could be used as a standalone intervention, an intervention for those on waiting lists for traditional therapy, as an adjunct to traditional therapy, or to facilitate treatment gains post-treatment.

The Course comprises material both in didactic form, that is, text based instructions and information, and case-enhanced learning examples. Case-enhanced learning is informed by principles of Social Cognitive Theory (SCT) [33] and uses educational stories that identify a problem and a solution which an example (i.e., a case) resolves for the learner. The use of cases to facilitate learning is thought to assist in learning, adherence and engagement [34] while reducing defensiveness [34], [35], [36].

Lesson 1 of the Wellbeing Course includes information about the prevalence of anxiety disorders and depression, their role in affecting achievement of life goals, and how cognitive, behavioural, and physical symptoms maintain poor emotional health. Lesson 2 describes strategies for developing realistic cognitions. Lesson 3 describes physical de-arousal strategies and strategies for re-engaging in reinforcing activities. Lesson 4 describes avoidance and safety behaviours and principles of graded exposure. Lesson 5 describes principles of relapse prevention.

Each lesson is presented in a slide format combining text and photos, with approximately 60 slides per lesson, and 50 words per slide. Automated analyses of readability indicate the text has a mean Flesch-Kincaid Grade level of 6.1, a Simple Measure of Gobbledygook (SMOG) Index of 7, and Automated Readability Index of 5.3, indicating suitability for 8–9 year olds. Participants are instructed to read lessons in order over 8 weeks. Lessons 1, 2, 3, 4, and 5 are available at the beginning of weeks 1, 2, 4, 5, and 7, respectively. This timetable provides participants with additional time for the most complex components of the intervention, namely skills for managing cognitive and behavioural symptoms. Recommended homework assignments are described in a *Do It Yourself Guide* for each lesson. Additional resources include materials about assertiveness, communication and problem solving skills, and sleep hygiene skills.

### Procedure

TG and TEG participants received an email at the start of the Course providing guidelines and a recommended timetable to get the most out of the Course. TEG participants also received at least two additional emails per week during the Course. Some emails were triggered based on participant behaviour: specifically, emails were triggered when (1) participants completed each Lesson during the Course, and (2) if participants had not completed a Lesson within 7 days of it becoming available. Emails were also triggered according to the Course timeline: specifically, emails were triggered (1) at the beginning of each week when new Lessons became available or, if no new Lessons became available, to suggest some tasks for the week, and (2) at set times when participants were known to experience increases in symptoms or to have increased difficulties practicing skills (e.g., during the weeks where graded exposure is introduced). The emails were written and designed to (1) make sure participants always new about new content available on the site, (2) remind participants about unread materials, (3) reinforce progress and skills practice, (4) ‘normalise’ the challenges of learning new skills, and (5) emphasise and explain that symptom reduction required gentle, but consistent, practice of the skills over time. Each email was brief and was comprised of 2 to 3 paragraphs containing 3 or 4 concise sentences. Each email used the participant’s first name and was written to convey a warm and supportive tone. All participants consented to receive the emails and no emails contained personal or detailed clinical information.

Controls completed pre-treatment questionnaires at the same time as the treatment groups, but had no further contact with the researchers until post-treatment. Control group participants then received the same post-treatment questionnaires as the treatment groups and began treatment as soon as these questionnaires were complete. The control group participants received the same treatment as the TEG.

### Statistical Methods

Participants who did not complete pre-treatment questionnaires were not included in any analyses. This was necessary as participants began the intervention in cohorts following randomisation. Group differences in demographic data and pre-treatment measures were analysed with one-way analysis of variance (ANOVAs) followed by *t*-tests with Bonferroni corrected *p* values and chi-square tests.

Two sets of analyses were conducted to examine the primary hypotheses. The mixed-models approach with an autoregressive covariance structured and using maximum likelihood estimation was identified as the best way to handle missing data at post-treatment and three month follow-up. The first set of analyses were conducted using the entire data set (i.e., the O*verall Sample*) and the second set of analyses were restricted to participants with elevated symptoms of both anxiety and depression (i.e., the *Comorbid Sample*), defined by pre-treatment scores above clinical cut-offs on both primary outcome measures (i.e., GAD-7≥8 and total PHQ-9≥10). Differences in questionnaire scores within and between groups at the different time points were examined using pairwise comparisons. Effect sizes (Cohen’s *d*) and 95% effect size confidence intervals were calculated both within-group and between-group effects based on the pooled standard deviation. All analyses were performed in SPSS version 19.0 (SPSS, Inc., Chicago, IL).

As described in recent dissemination studies [31], pre-treatment, post-treatment and follow-up PHQ-9 and GAD-7 scores were compared with clinical cut-offs to provide an index of clinically significant *remission*. This was defined as the proportion of participants who initially scored at or above the clinical cut-offs and then subsequently below the clinical cut-offs. Finally, the number of times safety protocols were triggered because of elevated PHQ-9 scores and the proportion of participants with significant deterioration in PHQ-9 or GAD-7 scores were reported. The latter was calculated for each group based on a completer analysis and defined as an increase in PHQ-9 or GAD-7 scores of 5 or more points from pre-treatment.

## Results

### Baseline Data

The mean age of participants was 41.30 years (SD = 9.76), and 186 (74.4%) were women. Additional demographic details are included in Table 1. A one way ANOVA and post-hoc tests revealed that Controls were older than TEG participants (*F_2, 254_* = 3.42, *P* = .034), and TG participants were less likely to be taking medication for mental health conditions than other participants (***χ*** **^2^** (2) = 6.20, *P* = .013). No other differences were found in demographic characteristics or in pre-treatment scores on the outcome measures (all *P*s>.05).

**Table 1:** Group demographic characteristics for the Overall Sample.

	*TEG (n = 100)*		*TG (n = 106)*		*Controls (n = 51)*		*Total*		Statistical Significance
Variable	*n*	%	*n*	%	*n*	%	*N*	%	
**Sex**									
Male	23	23	28	26.4	17	33.3	68	26.5	***χ*** **^2^** (2) = 1.85, *P* = 0.396
Female	77	77	78	73.6	34	66.7	189	73.5	
**Age**									
Mean	40.31 (10.13)	–	40.71 (9.07)	–	44.45 (9.97)	–	41.30 (9.76)	–	*F*_2,254_ = 3.42, *P* = 0.034
Range	18–59	–	20–58	–	22–59	–	18–59	–	
**Self-Identified Principal Problem**									
Depression	33	33.0	29	27.4	23	45.1	85	33.1	***χ*** **^2^** (6) = 9.69, *P* = 0.138
Generalised Anxiety Disorder	28	28.0	44	41.5	12	23.5	84	32.7	
Panic Disorder	17	17.0	11	10.4	6	11.8	34	13.2	
Social Phobia	22	22.0	22	20.8	10	19.6	54	21.0	
**Marital Status**									
Single/Never Married	31	31.0	25	23.6	12	23.5	68	26.5	***χ*** **^2^** (4) = 7.18, *P* = 0.127
Married/De Facto	57	57.0	70	66.0	27	52.9	154	59.9	
Separated/Divorced/Widowed	12	12.0	11	10.4	12	23.5	35	13.6	
**Education**									
None	1	1.0	0	0.0	0	0.0	1	0.4	***χ*** **^2^** (6) = 3.99, *P* = 0.677
High school	19	19.0	18	17.0	9	17.6	46	17.9	
Trade/Technical Certificate	21	21.0	15	14.2	8	15.7	44	17.1	
Diploma/Degree	59	59.0	73	68.9	34	66.7	166	64.6	
**Employment Status**									
Full time	49	49.0	49	46.2	18	35.3	116	45.1	***χ*** **^2^** (4) = 5.26, *p* = .325
Part time/Student	31	31.0	34	32.1	18	35.3	83	32.3	
Unemployed, Retired or Disabled	20	20.0	23	21.7	15	29.4	58	22.6	
**Previous Mental Health Treatment**	84	84.0	87	82.1	41	80.4	212	82.5	***χ*** **^2^** (2) = 0.326, *P* = 0.850
**Taking Medication for Mental Health Condition**	45	45.0	30	28.3	24	47.1	99	38.5	***χ*** **^2^** (2) = 8.02, *P* = 0.018

*Note.* TEG: Treatment Plus Automated Email Group. TG: Treatment Group.

### Adherence and Attrition

**Figure 1:**
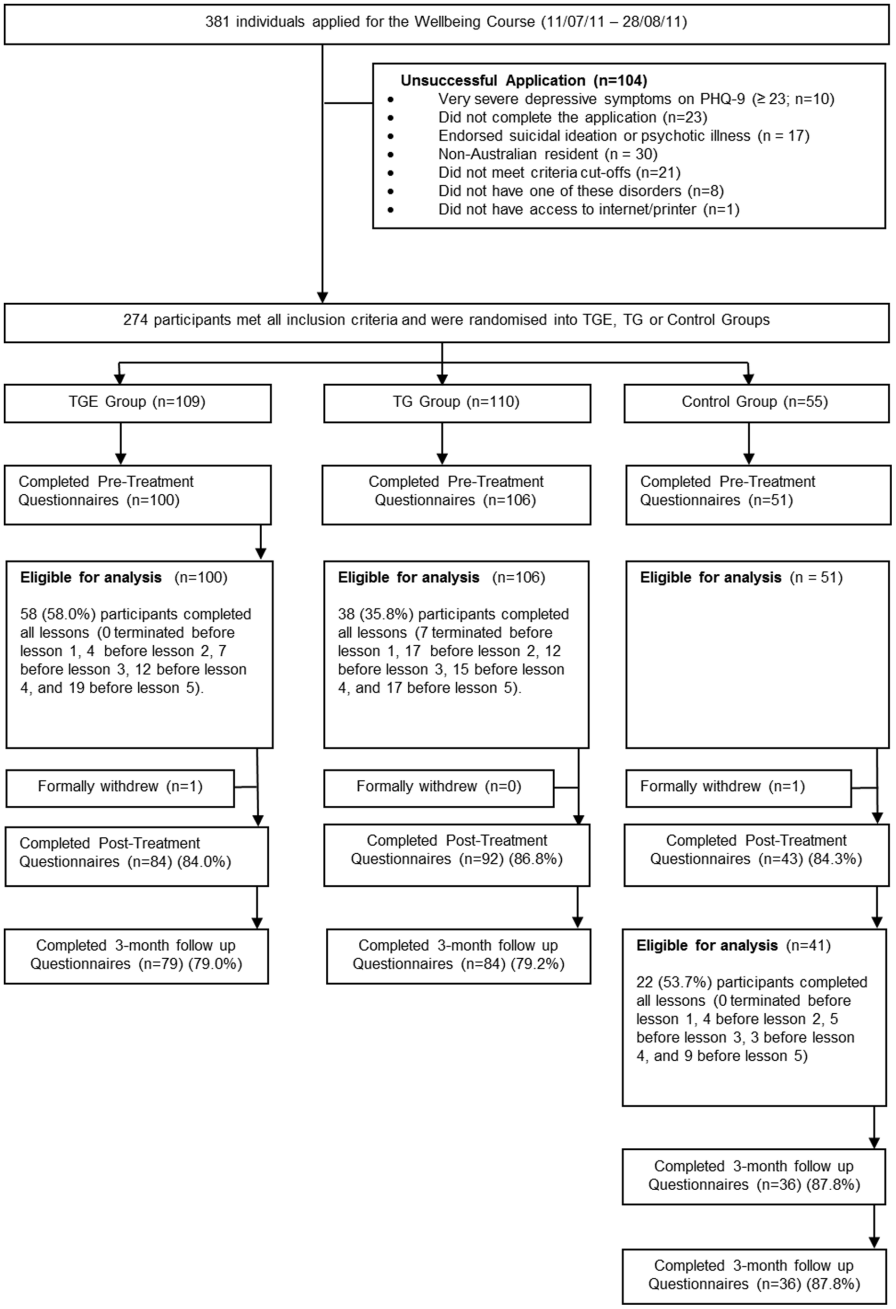
Wellbeing study flow chart. PHQ-9, Patient Health Questionnaire –9 Item. TEG: Treatment Plus Automated Email Group. TG: Treatment Group.

The flow is shown in Figure 1. More TEG participants (58.0%) completed the course, defined as reading the five lessons within the eight weeks, than TG (35.8%) participants (***χ*** **^2^** (1) = 10.15, *P* = .001) in the Overall Sample. A similar pattern was observed in the Comorbid Sample with more TEG participants (56.7%) completing the course than TG (29.6%) participants (***χ*** **^2^** (1) = 8.44, *P* = .004). As indicated in Figure 1, considerably more TG participants (34.0%) terminated before completing lesson 3 than TEG participants (11.0%). Completers did not differ from non-completers on either PHQ-9 or GAD-7 pre-treatment scores (*F_1, 204_* = 0.39–1.67, *P*>.05).

Post-treatment data were collected from 84.0% TEG, 86.8% TG, and 84.3% Control participants. Three-month follow-up data were collected from 79.0% TEG and 79.2% TG participants. ANOVAs revealed that those who did not complete follow-up questionnaires had significantly higher pre-treatment PHQ-9 scores (*F_1, 204_* = 9.33, *P* = .003) but not GAD-7 scores (*F_1, 204_* = 0.06, *P* = .80), than questionnaire completers.

### Primary Outcome Measures

**Figure 2:**
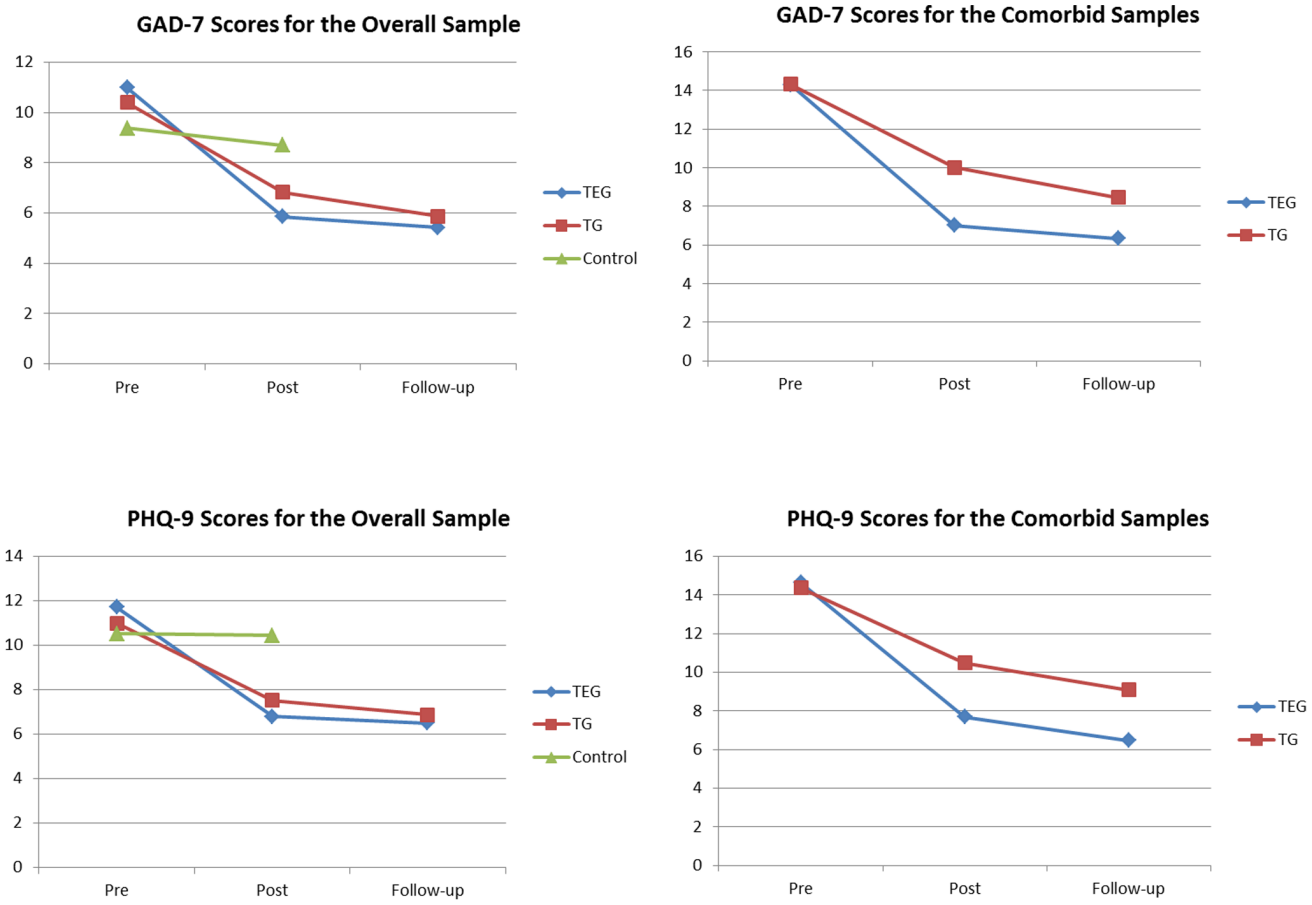
Means for the PHQ-9 and GAD-7 for the Overall and Comorbid Samples. Note: TEG: Treatment plus automated email group. TG: Treatment group. PHQ-9: Patient Health Questionnaire-9 Item. GAD-7: Generalised Anxiety Disorder-7 Item.

Means for the PHQ-9 and GAD-7 for the Overall and Comorbid Samples are included in Table 2. The means for the Control Group after they received the same treatment as the TEG are also shown in Table 2. Changes in PHQ-9 and GAD-7 scores for the Overall and Comorbid Samples are shown in Figure 2.

**Table 2:** Results of outcome measures for the Overall Sample: Observed and Estimated Means, standard deviations, confidence intervals and effect sizes (Cohen’s *d*) for each group.

	N	Observed Means			Estimated Means			Effect sizes (based on estimated means)				
		Pre	Post	Follow-up	Pre	Post	Follow-up	Within GroupPre-post	Post Between Group TEGvs. TG	Post Between Group vs. Control	Within Group Pre-follow	Follow-up Between Group TEG vs. TG
**PHQ-9**												
TEG	100	11.71 (4.72)	6.73 (4.50)	6.24 (4.42)	11.71 (4.95)	6.79 (5.31)	6.49 (5.35)	.96 (.66–1.25)	.14 (−.14–.41)	.68 (.33–1.03)	1.01 (.71–1.30)	.07 (−.20–.34)
TG	106	10.99 (4.92)	7.41 (5.91)	6.38 (5.07)	10.99 (4.95)	7.51 (5.28)	6.87 (5.34)	.68 (.40–.95)		.55 (.21–89)	.80 (.52–1.08)	
Control	51	10.53 (4.63)	10.50 (5.04)	–	10.52 (4.94)	10.44 (5.41)	–	.02 (−.37 –.40)	–	–	–	–
**GAD-7**												
TEG	100	10.98 (4.23)	5.75 (3.78)	5.51 (4.13)	10.98 (4.57)	5.85 (4.89)	5.42 (4.90)	1.08 (.78–1.38)	.20 (−.07–.47)	.58 (.23−.92)	1.17 (.87–1.47)	.09 (−.18–.36)
TG	106	10.40 (4.98)	6.80 (5.62)	5.60 (4.25)	10.39 (4.57)	6.83 (4.83)	5.87 (4.89)	.76 (.48–1.03)		.38 (.04–.72)	.96 (.67–1.24)	
Control	51	9.37 (4.39)	8.75 (4.44)	–	9.37 (4.56)	8.69 (4.98)	–	.14 (−.25–.53)	–	–	–	–
**Control (In** **Treatment)**												
GAD-7	42	8.76 (4.44)	5.58 (4.17)	4.53 (3.43)	8.76 (4.23)	5.63 (4.54)	4.74 (4.52)	.71 (.27–1.15)	–	–	.92 (.46 (1.36)	–
PHQ-9	42	10.50 (5.04)	5.69 (4.08)	5.67 (4.66)	10.50 (4.85)	6.05 (5.15)	6.11 (5.08)	.89 (.43–1.33)			.88 (.43–1.32)	

*Note.* Standard deviations and confidence intervals are shown in parentheses. TEG: Treatment Plus Automated Email Group. TG: Treatment Group; Pre: Pre-treatment, Post: Post-treatment; Follow-up: 3 month follow-up; PHQ-9: Patient Health Questionnaire 9-Item; GAD-7: Generalised Anxiety Disorder 7-Item.

#### Overall Sample

The mixed-models analyses examining PHQ-9 scores (Table 2) revealed a significant effect for Time (*F_2, 373_* = 61.66, *P*<.001) and a significant Time by Group interaction, (*F_3, 416_* = 6.79, *P*<.001), but no significant effect for Group (*F_2, 277_* = 1.882, *P* = .154). Pairwise comparisons revealed significant differences between TEG and Controls (*P*<.001) and between TG and Controls (*P* = .001) at post-treatment, but no significant difference between treatment groups at post-treatment (*P* = .333) or three month follow-up (P = .611).

Similarly, the analyses examining GAD-7 scores revealed a significant effect for Time (*F_2, 370_* = 105.07, *P*<.001) and a significant Time by Group interaction, (*F_3, 416_* = 7.43, *P*<.001), but no significant effect for Group (*F_2, 269_* = 0.487, *P* = .615). Pairwise comparisons revealed significant differences between TEG and Controls (*P* = .001) and between TG and Controls (*P* = .0028) at post-treatment, but no significant difference between treatment groups at post-treatment (*P* = .151) or three month follow-up (*P* = .505).

#### Comorbid Sample

The mixed-models analyses examining PHQ-9 scores (Table 2) for the Comorbid Sample revealed a significant effect for Time (*F_2, 158_* = 76.81, *P*<.001) and Group (*F_1, 107_* = 5.03, *P* = .027), and a significant Time by Group interaction, (*F_2, 158_* = 4.03, *P* = .020. Pairwise comparisons revealed that both groups improved significantly from pre-treatment to post-treatment (*P*s<.001), but not from post-treatment to follow-up (*P*>.099). However, the pairwise comparisons also revealed significant differences between TEG and TG Groups at post-treatment (*P* = .008) and three month follow-up (*P* = .015) with the TEG Group reporting lower symptoms on the PHQ-9 at both time points.

The analyses examining GAD-7 scores revealed a significant Time by Group interaction, (*F_2, 150_* = 3.78, *P* = .025) and significant main effects for Time (*F_2, 150_* = 106.34, *P*<.001) and Group (*F_1, 99_* = 5.51, *P* = .021). Pairwise comparisons revealed that both groups improved significantly from pre-treatment to post-treatment (*P*s<.001), but that the TG Group improved significantly from post-treatment to follow-up (*P* = .033) where the TEG Group showed no significant change in GAD-7 scores from post-treatment to follow-up (*P* = .320). The pairwise comparisons also revealed the TEG Group reported significantly lower symptoms than the TG Group at post-treatment (*P* = .002) and three month follow-up (*P* = .031).

### Effect Sizes

Within- and between-group effect sizes for the outcome measures are included in Tables 2 and 3. For both the Overall and Comorbid Samples large (≥0.8) within-group effect sizes were found for the TEG, and moderate (0.5<0.8) to large within-group effect sizes were found for the TG. Small (<0.5) between-group effect sizes were found between the two treatment groups, while moderate between-group effect sizes were found for the diagnostic samples.

**Table 3:** Results of outcome measures for the Comorbid Sample (cut-offs on both PHQ-9 and GAD-7): Observed and Estimated Means, standard deviations, confidence intervals and effect sizes (Cohen’s *d*) for each group.

	n	Observed Means			Estimated Means			Effect sizes (based on estimated means)			
		Pre	Post	Follow-up	Pre	Post	Follow-up	Within Group Pre-post	Post Between Group TEG vs. TG	Within Group Pre-follow	Follow-up Between Group TEG vs. TG
**PHQ-9**											
TEG	47	14.64 (3.34)	7.58 (4.60)	6.34 (4.58)	14.63 (4.61)	7.69 (4.95)	6.47 (4.99)	1.45 (.99–1.89)	.56 (.14–.96)	1.70 (1.21–2.16)	.51 (.09–.92)
TG	46	14.39 (3.33)	10.57 (6.16)	8.94 (6.27)	14.39 (4.61)	10.48 (5.10)	9.09 (5.28)	.80 (.37–1.22)		1.07 (.62–1.50)	
**GAD-7**											
TEG	47	14.30 (3.01)	6.70 (4.39)	6.39 (4.30)	14.29 (4.21)	7.02 (4.71)	6.34 (4.58)	2.02 (1.51–2.50)	.65 (.22–1.06)	2.05 (1.54–2.54)	.45 (.04–.86)
TG	46	14.33 (3.36)	10.16 (6.07)	8.50 (4.62)	14.32 (4.27)	10.01 (4.53)	8.46 (4.83)	.96 (.52–1.38)		1.29 (.83–1.72)	

*Note.* Standard deviations and confidence intervals are shown in parentheses. TEG: Treatment Plus Automated Email Group. TG: Treatment Group; Pre: Pre-treatment, Post: Post-treatment; Follow-up: 3-month follow-up; PHQ-9: Patient Health Questionnaire 9-Item; GAD-7: Generalised Anxiety Disorder 7-Item.

### Clinical Significance

#### Overall Sample

With respect to remission, significantly more TEG (55.9% and 57.9%) and more TG (53.7% and 56.8%) participants scored below the PHQ-9 and GAD-7 clinical cut-offs than Controls (24.1% and 21.9%) at post-treatment, respectively, with no difference between treatment groups (Table 4). These gains were sustained at follow-up, again with no difference between treatment groups.

**Table 4:** Proportion of participants in the Overall and Diagnostic Sample, by group, above and below cut-off scores of clinical significance (remission).

Overall Sample	TEG		TG		Control		Chi Square	
	n	%	n	%	n	%	Between group	TGE vs. TG
**PHQ-9**								
Pre-treatment score ≥10/Total	68/100	68.0	67/106	63.2	29/51	56.9	***χ*** **^2^** (2) = 1.84, *P* = 0.398	
Post-treatment score <10 (Remission)	38/68	55.9	36/67	53.7	7/29	24.1	***χ*** **^2^** (2) = 9.05, *P* = 0.011	***χ*** **^2^** (1) = 0.06, *P* = 0.802
Follow-up score <10 (Remission)	45/68	66.2	42/67	62.7	–	–		***χ*** **^2^** (1) = 0.18, *P* = 0.672
**GAD-7**								
Pre-treatment score ≥8/Total	76/100	76.0	74/106	69.8	32/51	62.7	***χ*** **^2^** (2) = 2.96, *P* = 0.228	
Post-treatment score <8 (Remission)	44/76	57.9	42/74	56.8	7/32	21.9	***χ*** **^2^** (2) = 13.20, *P* = 0.001	***χ*** **^2^** (1) = 0.02, *P* = 0.888
Follow-up score <8 (Remission)	45/76	59.2	44/74	60.3	–	–		***χ*** **^2^** (1) = 0.00, *P* = 0.975
**Diagnostic Sample**								
Pre-treatment PHQ-9 score ≥10 and GAD-7 score ≥8/Total	60/100	60.0	54/106	50.9	22/51	43.1	***χ*** **^2^** (2) = 4.14, *P* = 0.126	
Post-treatment PHQ-9 score <10 and GAD-7 score <8 (Remission)	28/60	46.7	21/54	38.9	4/22	18.2	***χ*** **^2^** (2) = 5.49, *P* = 0.064	***χ*** **^2^** (1) = 0.70, *P* = 0.402
Follow-up PHQ-9 score <10 and GAD-7 score <8 (Remission)	30/60	50.0	23/54	42.6	–	–		***χ*** **^2^** (1) = 0.63, *P* = 0.429

*Note.* TEG: Treatment Plus Automated Email Group. TG: Treatment Group.

#### Comorbid Sample

At post-treatment, significantly more TEG (46.7%) and TG (38.9%) participants were below both cut-offs than Controls (18.2%), with no difference between treatment groups (Table 4). These levels of remission were sustained at follow-up, again with no difference between treatment groups.

### Treatment Satisfaction

At post-treatment, 71/74 (95.9%) TEG and 76/80 (95%) TG participants reported it was ‘*worth their time doing the course’*, and 72/74 (97.3%) TGE and 74/79 (93.7%) TG participants reported they would ‘*recommend this course to a friend with anxiety or depression’*. Chi-squared analyses failed to reveal any differences in these satisfaction ratings (*χ* ^2^ (2–3) range = 0.76–2.82, *P*>.05).

### Safety

Participants’ responses to the PHQ-9 were automatically monitored and those whose PHQ-9 total scores increased from pre-treatment by more than 5 points and who also had a total score of ≥15, indicating the presence of moderately severe depression were sent an automated email providing instructions about how to contact crisis services in the event of a mental health emergency. Those whose PHQ-9 scores were above 20, indicating severe depression, or scoring ‘3’ to question 9 of the PHQ-9, were telephoned by the researchers who conducted an assessment and provided management. Five (5.0%) TEG, 1.9% TG, and no Controls received emails, and an additional 3.0% TEG, 2.8% TG, and 2.0% Control participants received both telephone calls and emails. The remainder of participants did not receive contact from the researchers until the 8 weeks had elapsed.

### Deterioration

#### Overall Sample

At post-treatment and follow-up up to 4.0% of TEG, 7.5% of TG and 13.7% of Control participants obtained PHQ-9 or GAD-7 scores five or more points higher compared to pre-treatment.

#### Comorbid Sample

At post-treatment and follow-up, up to 2.0% of TEG, 7.5% of TG and 5.9% of Control participants obtained PHQ-9 or GAD-7 scores five or more points higher compared to pre-treatment.

### Control Group Participants

Forty one of the 51 Control group participants elected to begin the Wellbeing Course after the Treatment Groups completed, and received the same treatment as the TEG. Twenty two of 41 (53.7%) Control Group participants completed the course within the 8 weeks, and post-treatment data and 3-month follow-up were collected from 87.8% and 87.8% participants, respectively.

The mixed-models analyses on the Control Group during treatment revealed significant main effects for Time on both the PHQ-9 (*F_2, 69_* = 21.11, *P*<.001) and GAD-7 (*F_2, 68_* = 14.55, *P*<.001). Pairwise comparisons revealed significant improvements on both the PHQ-9 and GAD-7 from pre-treatment to post-treatment (*P*<.001), but not post-treatment to 3-month follow-up (*P*>.257). These changes corresponded to effect sizes from pre-treatment to follow-up of 0.88 and 0.92 on the PHQ-9 and GAD-7, respectively.

With respect to remission, at post-treatment, 69.6% and 61.5% of Control Group participants scored below the PHQ-9 and GAD-7 clinical cut-offs, respectively, and these gains were sustained at follow-up. Ninety seven percent of Control group participants reported it was worth their time doing the Course, and 94% reported they would refer a friend.

## Discussion

The Wellbeing Course resulted in clinically significant reductions in symptoms of anxiety and depression related to a waitlist control group. These results indicated automated emails increased completion rates in a self-guided iPT intervention for people with anxiety and depression from 36% to 58%. No overall differences were found between participants who did and did not receive automated emails during the intervention in symptoms of anxiety or depression at post-treatment or follow-up. However, significant differences were found between participants who did or did not receive emails for elevated symptoms of both anxiety and depression. Specifically, those participants with elevated symptoms who received automated emails reported significantly less symptoms of anxiety and depression at post-treatment and follow-up compared with those who did not. These differences corresponded to moderate between-group effect sizes at post-treatment (*d* = 0.56 and 0.65) and follow-up (*d* = 0.45 and 0.51). Importantly, treatment satisfaction was high and the results were replicated in the Control Group, providing preliminary evidence for the reliability of automated emails in facilitating outcomes.

The present results are consistent with previous studies reporting the effectiveness of automated self-guided internet treatments for depression or anxiety, but are of a greater magnitude [12], [13], [15], [37]. These results also compare favourably with those obtained from trials of transdiagnostic iPT involving clinician guidance [7], [8], [9], trials of clinician-guided transdiagnostic interventions administered face-to-face [38], and with results from implementation trials in the UK IAPT service [31]. However, caution should be exercised in making comparisons across studies due to differences in samples, inclusion criteria, and methodologies.

The overall results indicate that adding automated emails can facilitate adherence and outcomes in self-guided internet-delivered interventions. These benefits are stronger for those with comorbid and more severe symptoms. Unfortunately, no firm conclusions can be drawn concerning how emails facilitated adherence or clinical outcomes in the present trial. However, the results parallel an extensive literature that indicates the benefits of minimal therapist contact added to self-help programs [39], [40]. Within this context therapists are likely to enhance motivation to continue in the course and engage in therapeutic activities. It is possible that automated emails serve the same motivational purpose and act in a similar fashion to a therapist, albeit in a less costly manner. If this is true, it would suggest that maximising the motivational efficacy or tailoring of automated emails might produce even stronger effects.

### Implications

These results indicate that, contrary to earlier reports [41], outcomes of self-guided internet-delivered interventions are beginning to approximate those obtained when support is provided by a therapist, at least, for populations attracted to online clinical trials. These results provide support for a model of psychotherapeutic change that emphasises the importance of systematically learning, practicing, and adopting core psychological skills that help to change unhelpful habits of thought and action. Systematically examining other strategies to promote engagement and adherence in self-guided iPT interventions is likely to further improve outcomes in iPT and will also have utility across a broad range of health conditions. We suspect the use of case-enhanced learning strategies in the course content facilitated engagement and adherence and, as noted by others, providing a clear deadline and expectations of feedback may also facilitate adherence and clinical outcomes [38].

Clinically effective self-guided iPT interventions are likely to be integrated with traditional models of care as therapists are more likely to refer consumers to self-guided interventions that are acceptable, engaging and effective. The results of the present study extend recent studies indicating that, following a therapist-administered structured diagnostic interview, consumers can obtain considerable benefit from a self-guided iPT intervention [17], [42], [43], [44]. Those who do not benefit from the self-guided intervention or who wish for more support can be subsequently referred for a more intense intervention. This simple stepped model has considerable potential for improving the capacity of existing mental health services, although needs to be tested.

Notwithstanding these encouraging findings, these results indicate that risk of deterioration is not insignificant. Between 5 and 8% of treatment group participants required intervention and, although this proportion will vary depending on the criteria used, they indicate that some people accessing publicly available websites will have significant psychological distress. Consequently, attention must be given to protocols for facilitating safety and referral to crisis services.

### Limitations

The generalisability of these findings needs to be considered in the context of the research design and participants. We aimed to simulate an intervention available on an open-access website. Thus, although participants applied independently and did not have contact with researchers until post-treatment, they were required to apply for the trial raising the potential of self-selected bias. However, a recent comparison of demographic and symptom profiles of participants in internet trials with those identified in epidemiological samples indicates the results of such trials may generalise to the wider population [45], although those results should be replicated with other samples. Unfortunately, no firm conclusions can be drawn concerning mechanisms through which emails facilitated outcomes and it is also notable that, in the overall sample, significant improvements in clinical outcomes were observed despite many participants not completing the whole Course. This is likely explained by the fact that, despite not completing the Course, most participants were exposed to significant levels of clinical content. Future research would, however, benefit from focusing on what level of clinical content is required to be reviewed or used in order to maximize and sustain clinical outcomes over the longer term.

While significant improvements were observed on symptoms of anxiety and depression, we did not conduct analyses on symptom measures relevant to specific anxiety disorders. In order to simulate an automated and publically available website the present study also had to rely on self-report measures. Thus, these results do not address questions concerning whether a transdiagnostic approach produces results comparable to disorder-specific interventions and future research may benefit from using non self-report based measures such as structured diagnostic interviews at follow-up time points. We have begun trials to explore and address both of these issues. In addition, the thresholds we used for triggering safety protocols and for determining clinically significant deterioration reflect the pragmatic requirements of this trial, and may not apply in other situations.

### Conclusions

The present results indicate that self-guided transdiagnostic iPT interventions can be developed, which result in clinically significant outcomes on generic measures of anxiety and depression and are acceptable to consumers. The inclusion of automated emails facilitated adherence and completion, particularly for those with comorbid or elevated symptoms. Replication is required with samples that include those with subclinical symptoms. Direct comparisons with disorder-specific programs are also necessary to determine the benefits of the Wellbeing Course for people with specific diagnoses. Further attention to developing strategies that facilitate adherence, engagement, learning and consumer safety are likely to help realise the potential of self-guided interventions.

## Supporting Information

Checklist S1. **CONSORT Checklist.**
(DOC)

Protocol S1 **Trial Protocol.**
(PDF)
