# Supplementary material for: Improving Adherence and Clinical Outcomes in Self-Guided Internet Treatment for Anxiety and Depression: Randomised Controlled Trial
Source: PLoS One. 2013 Jul 3;8(7):e62873. doi: 10.1371/journal.pone.0062873 (PMC3701078; doi:10.1371/journal.pone.0062873)
Supplement: Protocol S1 — Trial Protocol. (PDF) [file pone.0062873.s002.pdf]

Protocol Title

Randomized controlled trial of the effects of graded support on symptoms of anxiety and depression using an Internet-based education program for anxiety and depression

HREC Ref: TBA

Version Number: 1.0
Date of Protocol: 2/11/2010

SYNOPSIS
Protocol title: **Randomized controlled trial of the effects of graded support on symptoms of anxiety and depression using an Internet-based education program for anxiety and depression**

Protocol Version: 1.0

LIST OF INVESTIGATORS

Chief Investigator: N Titov
Address: Clinical Research Unit for Anxiety and Depression, School of Psychiatry, UNSW at St Vincent's Hospital
Telephone no.: +612 8382 1406
Fax no.: +612 8382 1401

Principal Investigator: B Dear
Address: Clinical Research Unit for Anxiety and Depression, School of Psychiatry, UNSW at St Vincent's Hospital
Telephone no.: +612 8382 1418
Fax no.: +612 83821401

Principal Investigator: J G Andrews
Address: Clinical Research Unit for Anxiety and Depression, School of Psychiatry, UNSW at St Vincent's Hospital
Telephone no.: +612 8382 1405
Fax no.: +612 83821401

Principal Investigator: J. Spence
Address: Clinical Research Unit for Anxiety and Depression, School of Psychiatry, UNSW at St Vincent's Hospital
Telephone no.: +612 8382 1430
Fax no.: +612 8382 1401

Associate Investigator: K Solley
Address: Clinical Research Unit for Anxiety and Depression, School of Psychiatry, UNSW at St Vincent's Hospital
Telephone no.: +612 8382 1416
Fax no.: +612 83821401

Associate Investigator: B Wootton
Address: Clinical Research Unit for Anxiety and Depression, School of Psychiatry, UNSW at St Vincent's Hospital
Telephone no: +612 8382 1421
Fax no.: +612 8382 1401

Associate Investigator: L Johnston
Address: Clinical Research Unit for Anxiety and Depression, School of Psychiatry, UNSW at St Vincent's Hospital
Telephone no: +612 8382 1417
Fax no.: +612 8382 1401

Summary

**Protocol title:**	Randomized controlled trial of the effects of graded support on symptoms of anxiety and depression using an Internet-based education program for anxiety and depression
**Protocol version:**	1.0
**Objectives:**	**Primary objectives:** To determine the benefits of different levels of support on the efficacy of an internet-delivered trans-diagnostic iCBT education program for anxiety and depression.<br>**Secondary objective:** To determine variables likely to facilitate completion rates, satisfaction, and acceptability to consumers.<br><br>The results will inform us about who is likely to benefit from a trans-diagnostic education protocol and how best to administer this program.
**Study design:**	A CONSORT-R compliant, registered RCT with 4 parallel groups (total sample size=350):<br>**Group 1: Waitlist control** (n=50). Receive access to the program once the other groups have completed the program (week 9).<br>**Group 2: Self-guided** (n=100). Receive immediate access to the program.<br>**Group 3: Self-guided + automatic emails** (n=100). Receive immediate access to the program.<br>**Group 4: Self-guided + automatic emails + weekly telephone support** (n=100). Receive immediate access to the program.
**Planned sample size:**	350
**Selection criteria:**	Australian adults aged 18-64 with elevated symptoms of depression and/or anxiety based on clinical measures.
**Study procedure:**	Recruit and treat participants with elevated symptoms of depression and/or anxiety using a 5-lesson Internet-based education program. Three groups begin the program immediately, with graded levels of support (none vs. automatic emails vs. automatic emails + weekly phone calls). Their results are compared with a waitlist control condition.
**Stat considerations**:	The design is powered to detect a between-groups effect size difference of 0.35 (alpha of 0.05, and power of 0.80) between Groups 2, 3, and 4. The design is also powered to detect a between-groups effect size difference of 0.55 between Group 1 and Groups 2, 3, and 4. Analyses will be conducted using intention-to-treat and completer models. Categorical data will be analysed via chi-squared tests and continuous data via parametric or equivalent non-parametric tests, including ANCOVAs and mixed linear models.
**Duration of the Study:**	23 months

TABLE OF CONTENTS PAGE

1. BACKGROUND	5
1.1 Disease Background	5
1.2 Rationale for Performing the Study	5
2. STUDY OBJECTIVES	
2.1 Primary Objective	6
2.2 Secondary Objective	7
3. STUDY DESIGN	
3.1 Design	7
3.2 Study Groups	7
3.3 Number of Participants	7
3.4 Number of Centres	7
3.5 Duration	7
4. PARTICIPANT SELECTION	
4.1 Inclusion Criteria	7
4.2 Exclusion Criteria	8
5. STUDY OUTLINE	
5.1 Study Flow Chart	9
5.2 Investigation Plan	10
5.3 Study Procedure Risks	11
5.4 Recruitment and Screening	12
5.5 Informed Consent Process	13
5.6 Enrolment Procedure	13
5.7 Randomisation Procedure	13
6. SAFETY	
6.1 Adverse Event Reporting	13
6.2 Serious Adverse Event Reporting	13
6.3 Participant Safety	13
6.4 Data Safety and Monitoring Board - membership and responsibilities	13
6.5 Early Study Termination	14
7. BLINDING AND UNBLINDING	14
8. STATISTICAL CONSIDERATIONS	14
8.1 Sample Size Calculation	14
8.2 Analysis Plan	14
9. STORAGE AND ARCHIVING OF STUDY DOCUMENTS	14
10. REFERENCES	15
11. APPENDICES	17
11.1 Questionnaires	18
11.2 Diagnostic Interview	27

1. BACKGROUND

1.1. Disease Background

Depression and anxiety are the most common mental disorders, frequently co-occur, and are associated with considerable distress and disability. However, both the 1997 and 2007 Australian National Surveys of Mental Health and Wellbeing reported that less than 40% of those with anxiety or depression sought treatment each year and of those, less than 50% receive evidence-based treatments [1-2].

1.2. Rationale for Performing the Study

The planned project is part of a larger research program exploring the potential of Internet-based education and treatment programs for anxiety and depression. Since 2007 we have conducted 20 RCTs of Internet cognitive behavioural therapy (iCBT) programs for people with anxiety disorders (generalized anxiety disorder, social phobia, or panic disorder) or depression. This research program has generated more than 30 publications, all RCTs have been approved or ratified by the UNSW and SVH HRECs, and all RCTs have resulted in clinical outcomes at least comparable to treatment outcomes we obtain from our face-to-face clinic (the Anxiety Disorder Clinic at St Vincent's Hospital, Sydney). Importantly, these results are also at least as good as those reported in recent meta-analyses of iCBT for depression and anxiety [3-4].

We have also recently completed 3 RCTs of Internet transdiagnostic treatment partly funded by an NHMRC Project Grant (#630560), again with good clinical outcomes and high levels of acceptability. Transdiagnostic programs treat anxiety and depression in the same program, and are designed to address the common issue of psychiatric co-morbidity, that is, patients having symptoms of more than one type of anxiety or depressive disorder [5]. Because anxiety disorders and anxiety and depression frequently co-occur, transdiagnostic programs offer practical advantages in terms of the delivery and receipt of education or treatment; that is, one program rather than several.

Our programs, while effective, have mostly been tested in trials that include guidance provided by a clinician or technician. Important questions are: **What types of support are required to achieve positive outcomes, and can adequate gains be obtained from a brief self-guided education program?** An effective self-guided trans-diagnostic iCBT program would offer multiple additional advantages: It would be highly cost-effective; scalable; would improve access to evidence-based education and treatments; and would teach consumers practical strategies for managing their own mental health. Until recently, self-guided Internet programs for anxiety or depression have been associated with small benefits [6-8]. More recent self-guided programs for depression [9] and panic disorder [10] indicate greater benefits are possible. Our own studies of self-guided programs to treat social phobia have demonstrated that the addition of regular automatic emails (providing encouragement and notifying when additional resources were available) doubled completion rates, and significantly improved clinical outcomes, and satisfaction rates [11-12], resulting in effect sizes of 1.0 on measures of social phobia. We have replicated this finding in social phobia, but note that this has not been replicated in other disorders.

This study will recruit 350 Australian adults (aged 18-64 years) with elevated symptoms of anxiety or depression. These participants will be given access to the "Wellbeing Program", a 5 lesson iCBT program they will be invited to complete over 8 weeks. This program has been carefully developed using evidence-based CBT techniques, parts have been tested in over

20 RCTs, and consultations have been conducted with more than 1000 consumers. This 5-lesson version has been successfully tested in a pilot study but not in an RCT.

Program Content: Components of this program include:

- 5 online lessons that include illustrated stories about adults who learn to manage symptoms of anxiety and depression
- Homework assignments
- Additional downloadable printed material describing techniques for managing other symptoms, including sleep difficulties, communication skills, and healthy lifestyle changes
- Participants may have full access to the program materials (Group 2: Self-Guided Group), full access + automatic emails (Group 3: Self-Guided + Automatic Emails Group), or full access + automatic emails + telephone support (Group 4: Weekly Support + Automatic Emails Group). The Waitlist Control Group (Group 1) will have access to the Self-Guided + Automatic Emails Group and will begin the program at week 9, once the education groups (Groups 2, 3, & 4) have completed.

Outcome Measures: We will measure changes in symptom level at the beginning of the 8 week program course of education, one week after the conclusion of education, at 3 month follow up, and at 12 month follow-up. The outcome measures will be the:

- Patient Health Questionnaire-9 Item (PHQ-9) [13]: Measures symptoms of depression.
- Generalized Anxiety Disorder-7 Item Scale (GAD-7) [14]: Measures symptoms of anxiety.
- Depression, Anxiety and Stress Scales-21 Item (DASS-21) [15]: Measures symptoms of anxiety, stress, and depression.
- The Panic Disorder Severity Rating Scale-Self Report [16]: Measures symptoms of panic disorder.
- The Social Phobia-12 [17]: Measures symptoms of social phobia.
- World Health Organisation Disability Assessment Schedule – 2nd Edition (WHODAS-II) [18]: Measures symptoms of disability.
- Kessler-10 Item (K-10) [19]: Measures symptoms of psychological distress.
- NEO-Five Factor Inventory – Neuroticism Subscale (NEO-FFI-NI) [20]: Measures personality features believed to underlie anxiety and depressive symptoms.

In addition, the GAD-7 and PHQ-9 will be administered at the beginning of each lesson to monitor anxiety and mood. We will also encourage feedback from participants at every stage of the project to determine how to improve the content and delivery of the program.

2.STUDY OBJECTIVES

2.1. PRIMARY OBJECTIVES AND HYPOTHESES AND ENDPOINTS

To determine the benefits of different levels of support on the efficacy of an internet-delivered trans-diagnostic iCBT education program for anxiety and depression, using a 4-group CONSORT-R compliant, registered RCT.

The main hypotheses are that

1. Improvements will be observed at Primary Endpoint (post-education) in symptoms of anxiety and depression: Groups 4>3>2>1
2. Differences will be observed in the acceptability of the program: Groups 3>2>1
3. Differences will be observed in completion rates: Groups 3>2>1

The Primary Endpoint coincides with the post education questionnaires at week 9.

The Secondary Endpoint occurs at 3-month follow-up.
The Tertiary Endpoint occurs at 12-month follow-up.

2.2. SECONDARY OBJECTIVES

To identify variables likely to facilitate completion rates, satisfaction, and acceptability to consumers. To identify characteristics of people unlikely to benefit.

The results will inform us about who is likely to benefit from a trans-diagnostic education protocol and how best to administer this program in the future.

3.STUDY DESIGN

3.1. DESIGN

CONSORT-R compliant 4 parallel-group Randomised Controlled Trial

3.2. STUDY GROUPS

4 parallel groups (total sample size=350):

1. Waitlist control (n=50). Receive access to the program once the other groups have completed the program (week 9).
2. Self-guided (n=100). Receive immediate access to the program.
3. Self-guided + automatic emails (n=100). Receive immediate access to the program.
4. Self-guided + automatic emails + weekly telephone support (n=100). Receive immediate access to the program.

3.3. NUMBER OF PARTICIPANTS

350 in total: n=50 in Group 1, and 100 in Groups 2-4.

3.4. NUMBER OF CENTRES

1 – Clinical Research Unit for Anxiety and Depression, St Vincent's Hospital, Sydney

3.5. DURATION

1 February 2011 to 31 December 2012, inclusive of education, follow up and write up of results. The Primary Endpoint will be the end of active education for Groups 2-4 (who receive education immediately, i.e. 9 weeks after beginning the program), the Secondary Endpoint will be the 3 month follow up of these participants (i.e., 3 months after completion of education), and the Tertiary Endpoint will be the 12 month follow-up of these participants (i.e., 12 months after completion of education).

4. PARTICIPANT SELECTION

4.1. INCLUSION CRITERIA

1. Elevated symptoms of depression or anxiety as indicated by: Total scores on at least one of the following measures: PHQ-9 > 9; GAD-7 > 7; Mini SPIN > 5 [21]; ANSQ > 1 [22]. Scores above these cut-offs indicate likelihood of meeting diagnostic criteria for at least one of: depression, generalized anxiety disorder, social phobia, panic disorder, respectively.
2. Self-identifying as having difficulties with either depression, social phobia, panic disorder, or generalized anxiety disorder. Note that meeting diagnostic criteria for a formal anxiety disorder or depression is NOT an inclusion criteria – instead, participants need to have elevated symptoms of anxiety or depression as indicated in (1), above.
3. No current participation in cognitive behavioural treatment.
4. No change in medications in the period 1 month prior to this study, and no change in medications during the study.
5. Access to the Internet and a printer.

6. 18 years to 64 years old.
7. Resident of Australia.
8. Prepared to provide name, phone number and address, and to provide the name and address of a local general practitioner
9. Prepared to provide written informed consent.

4.2. EXCLUSION CRITERIA

1. Severe depression (score of 23 or greater on PHQ-9)
2. Suicidal intent or plan (either reporting suicidal intent or responding >2 to Question 9 [suicidal ideation] on the PHQ-9)
3. Scoring below cut-offs on the measures of anxiety and depression
4. Current substance abuse
5. Psychosis
6. Primary presenting problem not depression, generalized anxiety disorder, social phobia, or panic disorder.

5.STUDY OUTLINE

5.1. Study Flow Table

**Date**	**Stage**	**Groups**			
**February-March 2011**	**Recruitment**	**Eligible applicants randomized to:**			
		**Group 1 Waitlist Control (n=50)**	**Group 2 Self Guided (n=100)**	**Group 3 Self Guided + Auto Emails (n=100)**	**Group 4 Self Guided + Auto Emails + Weekly Support (n=100)**
**April 2011**	**PRE-EDUCATION** (administer questionnaires + diagnostic interview)	**Waitlist**	**Begin education**		
**April-May 2011**	**EDUCATION**	**Waitlist**	**Receive education**		
**June 2011**	**POST-EDUCATION Primary Endpoint** (administer post-education questionnaires + diagnostic interview)	Waitlist (administer questionnaires + diagnostic interview) **Begin education**	**POST-EDUCATION Primary Endpoint** (administer post-education questionnaires + diagnostic interview)	**POST-EDUCATION Primary Endpoint** (administer post-education questionnaires + diagnostic interview)	**POST-EDUCATION Primary Endpoint** (administer post-education questionnaires + diagnostic interview)
		Receive education	**Follow-up period**		
**September 2011**	**3-MTH FOLLOW-UP Secondary Endpoint** (administer questionnaires)	**POST-EDUCATION** (administer questionnaires)	**3-MTH FOLLOW-UP Secondary Endpoint** (administer questionnaires)		
		Write up Results			
**June 2012**	**12-MTH FOLLOW-UP Tertiary Endpoint** (administer questionnaires + diagnostic Interview)	No follow-up	**12-MTH FOLLOW-UP Tertiary Endpoint** (administer questionnaires + diagnostic Interview)		
		Write up 12 month follow-up			

5.2. INVESTIGATION PLAN

A CONSORT-R compliant, registered RCT comparing 4 parallel groups (total n = 350).

This design tests the relative benefits of different levels of support on symptoms of depression and anxiety and will help determine the acceptability of the program, and whether the education program is superior to natural remission. The 3 groups who immediately receive education (Groups 2, 3, & 4) will obtain different levels of support, compared to the fourth group (Group 1) a waitlist control (delayed education) group. As in any RCT accurate patient selection, good randomization, reliable and valid outcome measures, and low drop-out rates are critical. Analyses based on completers and on intention-to-treat will be performed.

Patients will be randomly allocated to one of the four groups (total n=350). Randomization will be done via www.random.org (using a sequence generated by an overseas colleague not associated with this research).

1. **Group 1: Waitlist control** (n=50). This group will receive access to the entire program + automatic emails once the other groups have completed the program (week 9).
2. **Group 2: Self-guided** (n=100). This group will receive immediate access to the entire program.
3. **Group 3: Self-guided + automatic emails** (n=100). This group will receive immediate access to the program + will receive regular automatic emails. These emails provide notification of when additional resources become available and help remind the participant about tasks to practice.
4. **Group 4: Self-guided + automatic emails + weekly telephone support** (n=100). This group receive immediate access to the program + regular automatic emails, and will also receive a brief weekly telephone call from one of the researchers. The aim of this weekly telephone contact is to reinforce progress, provide guidance about aspects of the program the participant may not be aware of, and to provide encouragement.

The education program is delivered via the website www.virtualclinic.org.au. The program consists of 5 online lessons conducted over an 8 week period, in which participants are encouraged to complete one lesson every 10 days. The program is comprised of the following lessons and the materials include key components of CBT for anxiety and depression:

1. *Lesson One:*
    a. Learn about the physical, cognitive and behavioural symptoms of anxiety and depression
    b. Learn about the principles of CBT

2. *Lesson 2:*
    a. Learn about the relationship between the way you think and feel
    b. Begin monitoring and challenging unhelpful thinking patterns

3. *Lesson 3:*
    a. Learn about physical symptoms of anxiety and depression and managing these with de-arousal and activation techniques
    b. Assertiveness skills

4. *Lesson 4:*
    a. Learn about behaviours (low activity and avoidance) that make anxiety and depression worse, and how to challenge those behaviours

5. *Lesson 5:*
    a) Learn about problem solving, relapse prevention and staying well

On application, applicants will complete screening measures of anxiety and depression. Those with scores too high or too low (asymptomatic) will not be eligible. Eligible applicants will be telephoned by

the researchers, who will conduct diagnostic interviews via telephone. Applicants who meet diagnostic criteria for a primary disorder other than those targeted by this education protocol will not be eligible but will be informed about other options for education or treatment.

At the beginning of the education program all participants will complete the outcome measure questionnaires. The questionnaires being administered in this study are all valid and reliable clinical and research measures.

All participants will complete a second entire set of questionnaires at the post-education point (Primary Endpoint) and will also be telephoned by the researchers who will conduct the telephone interviews again. Participants in Groups 2, 3 and 4 will also be asked to complete a shorter set of questionnaires at 3 and 12 months follow-up. Participants in Group 1 (Waitlist control group) will begin the program immediately after the other groups have completed the education.

In addition, brief measures of anxiety and depression (the GAD-7 and PHQ-9) will be administered at the beginning of each lesson to monitor anxiety and mood. We will also encourage feedback from participants at every stage of the project to determine how to improve the content and delivery of the program.

5.3. STUDY PROCEDURE RISKS

It is expected that adults with symptoms of depression and anxiety will experience clinically significant reductions in the severity of their symptoms. It is also hoped that the techniques taught in this program will be applied by participants after the trial is completed, resulting in long term improved management of symptoms of anxiety. However, because anxiety and depression often develop in teenage hood and early adulthood, it is likely that most of participants will have experienced distressing levels of emotions for several decades. The education protocol encourages them to learn and practice techniques for managing symptoms of anxiety and depression and, in the short term, this often leads to an increase in levels of anxiety.

Our priority is to support participants to stay safe. Risk management will occur at all stages of this project including:

At Application:
Applications with severe symptoms of depression (total score of >23 on the PHQ-9, a reliable and valid measure of the symptoms of depression), will not be eligible to participate, and will be provided with written contact details of how to access urgent support and will be strongly encouraged to do so. Applicants assessed as having a primary disorder not targeted by this program (as determined by diagnostic telephone interview) will not be eligible for this program, but will be informed about more appropriate iCBT program which target their specific problem, as well as being encouraged to consider other education and treatment options including accessing their GP or local services.

All applicants will also be screened for risk of suicide or self harm. Those at risk will not be eligible to participate, and will be provided with written contact details of how to access urgent support and will be strongly encouraged to do so.

All applicants will be informed (verbally and in writing) of steps to take if they feel emotionally distressed during the trial, including the contact details of the researchers, the importance of contacting their own GP, and contact details of emergency services. Applicants must also provide contact details for their GP, who we will only contact if we are sufficiently concerned about the participant (this is explained to participants verbally, and in the Participant Information Sheet). We do not contact GPs as a standard protocol, as many participants have said that they prefer their participation to remain confidential.

During the Trial:
All participants will be screened regularly for deterioration in mood or distress when they complete brief measures of anxiety and depression (prior to completing each new lesson). All electronic communications (emails) contain contact details for emergency services (in the footer), and written details about staying safe in mental health emergencies are included in several locations in the program materials.

During Completion of Questionnaires:
Questionnaires of anxiety and mood will be completed at pre-education, prior to completing each new lesson, at post-education, at 3 month follow-up, and for Groups 2, 3, and 4, also at again at 12 month follow-up.

Any deterioration in mood or elevated distress will trigger protocols involving contacting the participant to discuss their emotional state, and a meeting between the Clinical Psychologist and Chief Investigator/other researchers to develop a management plan, which is then documented and followed up as per VirtualClinic policy.

Members of the research team will meet at least 2 X weekly to discuss progress of each participant and of the overall trial. The Chief Investigator will monitor overall progress weekly and will take overall responsibility for this trial. All communications with participants will be documented as per VirtualClinic documentation policies. All adverse events will be promptly reported to the HRECs and CRUfAD Director.

5.4. Recruitment and Screening

Recruitment
Recruitment involves the following steps:

1. The study will be described on the intake website, www.virtualclinic.org.au. People will read the information about the study and elect to continue or not. If they choose to apply for the study they will complete an automated screening questionnaire to screen out participants with a history of schizophrenia or bipolar disorder, active suicidality, severe symptoms of depression, current substance use/dependence, or those who are asymptomatic.

2. People whose responses to the questions meet selection criteria will be telephoned by the researchers at a time convenient to the participant to administer a diagnostic interview (the Mini International Neuropsychiatric Interview Version 5.0.0 (MINI)) [23] to determine whether the participant meets criteria for an anxiety disorder. Note that meeting diagnostic criteria for a formal anxiety disorder or depression is NOT an inclusion criterion – instead, participants need to have elevated symptoms of anxiety as measured by outcome questionnaires.

3. Applicants assessed at diagnostic telephone interview as having a primary diagnosis of post-traumatic stress disorder, obsessive compulsive disorder or another disorder not targeted by this education protocol will not be eligible for this program, but will be informed about other more appropriate iCBT programs as well as being encouraged to consider other education and treatment options including accessing their GP or local services.

4. Those who meet the inclusion criteria will be randomly allocated to one of the four groups.

5. Participants will be sent the Participant Information Sheet and Consent Form via email and will return an electronic consent form via email to the investigators.

5.5. Informed consent process

Only participants who have read the information about the studies, elected to complete the screening questionnaire and meet inclusion criteria, will be invited to participate. A Participant Information Form and Consent Form will then be emailed to the participant. Informed consent will be taken as successfully returning the Consent Form. Additionally, informed consent will also be monitored during the study by the Chief Investigator; each time a patient logs on to do a lesson will be taken as a sign of continued consent to participate.

5.6. Enrolment procedure

Once participants have been accepted into the study they will receive an email confirming that they have been accepted pending return of their Consent Form. Once they have returned their Consent Form they will receive another email confirming this receipt and confirming the start date of the program.

5.7. Randomization procedure

The website www.random.org will be used to determine a list of randomly generated numbers that correspond to a group. Such a list will be generated by an overseas colleague. Participants will be randomly allocated to one of the four groups after they complete the diagnostic interview.

6.SAFETY

6.1. Adverse event reporting

Regular team meetings (at least 2 times a week) will be conducted to monitor any difficulties participants may be having and ways of best dealing with these difficulties. Adverse events will be reported to the Ethics Committee.

6.2. Serious adverse event reporting

Serious adverse events will be reported to the HRECs via SAE documentation and emails. Such information will also be reported to the CRUfAD Director, Professor Gavin Andrews, and discussed at additional meetings of the researchers. All researchers work at the same site (St Vincent's Hospital), facilitating the ability to hold urgent or unplanned meetings.

6.3. Participant safety

During the trial all participants will be screened before completing each lesson when they complete brief measures of anxiety and depression. All electronic communications (emails) contain contact details for emergency services (in the footer), and written details about staying safe in mental health emergencies are included in several locations in the program materials.

Questionnaires of anxiety and mood will be completed at pre-education, before each of the 5 lessons, and at 3-month and 12-month follow-up. Any deterioration in mood or elevated distress triggers protocols involving contacting the participant to discuss their emotional state, and a meeting between the Clinical Psychologist and Chief Investigator/Other researchers to develop a management plan, which is then documented and followed up as per VirtualClinic policy. Participant safety and wellbeing is the paramount consideration. Participants will be invited to contact the investigators at any time should their wellbeing deteriorate.

6.4. Data safety and monitoring board

Data safety will comply with the National Statement on Ethical Conduct in Human Research [24]. Only the responsible clinicians, delegated research assistants, and Chief Investigator will see identified data. To maintain participant privacy and confidentiality, participants will not be able to use an alias/username that contains identifiable information (e.g real name). If they choose an alias/username that contains identifiable information, they will be requested to change it before beginning the Program.

6.5. Early termination

If a patient wishes to withdraw from the study once it has started, he or she can do so at any time without having to give a reason. This process will be supervised by the Chief Investigator. Upon early withdrawal, all participants will be provided with encouragement to access additional services via their GP. Additionally, all participants will be given the opportunity to provide feedback about their current symptoms. If participants withdrawing from the program do report a deterioration of their symptoms, they will be contacted by the Clinical Psychologist or Chief Investigator to discuss access to additional services. If participants do not choose to provide feedback about their symptoms, but the investigators have concerns about the participant's condition, we do have consent from all participants to contact their GP. This process will be supervised by the Chief Investigator.

7. BLINDING AND UNBLINDING

Study is not blinded.

8.STATISTICAL CONSIDERATIONS

8.1. Sample Size Calculation

The design is powered to detect a between-groups effect size difference of 0.35 (alpha of 0.05, and power of 0.80) between Groups 2, 3, and 4, requiring 100 participants in Groups 2, 3, and 4. The design is also powered to detect a between-groups effect size difference of 0.55 between Group 1 and Groups 2, 3, and 4, requiring 50 participants in Group 1.

8.2. Analysis Plan

Analyses will be conducted using intention-to-treat and completer models. Categorical data will be analysed via chi-squared tests, and continuous data via parametric or equivalent non-parametric tests including ANCOVAs and mixed linear models.

9.STORAGE AND ARCHIVING OF STUDY DOCUMENTS

Throughout the study, research investigators will have access to identified data in order to monitor participant progress. During the data analysis phase, re-identifiable data (i.e. coded data) will be used. At study completion, non-identifiable data will be written to a password protected database. The VirtualClinic has consulted with the AMA regarding transmission of medical data through the internet. To maintain patient privacy the VirtualClinic operates using a Secure Socket Layer to provide secure encryption of the VirtualClinic data. Additionally the database is firewall protected. Staff associated with the project are aware of, and will adhere to, the National Statement on Ethical Conduct in Human Research [51].

10.REFERENCES

1. Australian Bureau of Statistics. *Australian Social Trends.* June, 2009.
2. Andrews, G., & Titov, N. (2007). Depression is very disabling. The Lancet, 370(9590), 808-809.
3. Cuijpers P, Marks IM, van Straten A, Cavanagh K, Gega L, et al. (2009). Computer-aided psychotherapy for anxiety disorders: a meta-analytic review. *Cognitive Behavioural Therapy*, 38:66-82.
4. Andersson G, Cuijpers P (2009). Internet based and other computerized psychological treatments for adult depression: a meta-analysis. *Cognitive Behavioural Therapy*, 8:196-205.
5. Barlow, D. H., Allen, L. B., & Choate, M. L. (2004). Toward a unified treatment for emotional disorders. *Behavior Therapy, 35,* 205-230.
6. Christensen H, Griffiths KM, McKinnon A, Brittliffe K. Online randomized controlled trial of brief and full cognitive behavioural therapy for depression. *Psychol Med* 2006; 36:1737-1746
7. Clarke G, Reid Ed, Eubanks D, *et al*. Overcoming Depression on the Internet (ODIN): a randomized controlled trial of an Internet depression skills intervention program. J Med Internet Res 2002; 4(3):e14. Available: http://www.jmir.org/2002/3/e14/ via the Internet. Accessed 30 Nov 2009.
8. Clarke G, Eubanks D, Reid E, *et al*. Overcoming Depression on the Internet (ODIN) (2): a randomized trial of a self-help depression skills program with reminders. J Med Internet Res 2005; 7(2):e16. Available: http://www.jmir.org/2005/2/e16/ via the Internet. Accessed 30 Nov 2009.
9. Meyer B, Berger T, Caspar F, *et al.* Effectiveness of a novel integrative online treatment for depression (Deprexis): randomized controlled trial. J Med Internet Res 2009; 11:e15.
10. Klein B, Shandley K, Austin D, Nordin S. (2008). A Pilot Trial of 'Panic Online' as a Self-Guided Treatment for Panic Disorder. *E-Journal of Applied Psychology* [Online] 2008 Dec 23. [cited 03/02/2009]; 4:2. Available: http://ojs.lib.swin.edu.au/index.php/ejap/article/view/136.
11. Titov N, Andrews G, Choi I, *et al*. Randomized controlled trial of web-based treatment of social phobia without clinician guidance. Aust N Z J Psychiatry 2009; 43:913-919.
12. Titov N, Andrews G, Schwencke G, *et al*. An RCT comparing effect of two types of support on severity of symptoms for people completing Internet-based cognitive behaviour therapy for social phobia. Aust N Z J Psychiatry 2009; 43:920-926.
13. Kroenke K, Spitzer RL, Williams JBW. The PHQ-9. Validity of a brief depression severity measure. *J Gen Intern Med* 2001; 16:606–613.
14. Löwe B, Decker O, Müller S, Brähler E, Schellberg D, Herzog W, & Herzberg P Y. (2008). Validation and standardization of the Generalized Anxiety Disorder Screener (GAD-7) in the general population. *Medical Care, 46,* 266-274.
15. Lovibond SH, & Lovibond PF. (1995). *Manual for the Depression Anxiety Stress Scales*. *(*2nd ed.*)*. Sydney, Australia: Psychology Foundation.
16. Houck, P. R., Spiegel, D. A., Shear, M. K., & Rucci, P. (2002). Reliability of the self-report version of the panic disorder severity scale. *Depression and Anxiety, 15,* 183-185.
17. Peters L, Sunderland M, Andrews G, Rapee RM, Mattick RP. Development of a short form Social Interaction Anxiety and Social Phobia scale using nonparametric item response theory: The Social Phobia-12 (SP-12). Psychological Assessment. Submitted for publication
18. World Health Organization. World Health Organisation Disability Assessment Schedule 2nd Edition. Available from URL: http://www.who.int/icidh/whodas/.
19. Kessler RC, Andrews G, Colpe LJ, Hiripi E, Mroczek DK, Normand SL., et al. (2002). Short screening scales to monitor population prevalences and trends in non-specific psychological distress. Psychological Medicine, 32, 959-976.
20. Costa PT, & McCrae RR. (1985).*The NEO Personality Inventory Manual*. Odessa, FL: Psychological Assessment Resources.
21. Seeley-Wait, E., Abbott, M.J., & Rapee, R.M. (2009). Psychometric properties of the Mini-Social Phobia Inventory. Journal of Clinical Psychiatry, 11, 231-236.
22. Stein, MB, Roy-Byrne PP, McQuaid JR, et al. (1999). Development of a brief diagnostic screen for panic disorder in primary care. Psychosomatic Medicine, 61, 359-364.
23. Sheehan, D. V., Lecrubier, Y., Sheehan, K. H., Amorim, P., Janavs, J., Weiller, E., Hergueta, T., Baker, R., & Dunbar, G. C.. (1998). The Mini-International Neuropsychiatric Interview (M.I.N.I.):

The development and validation of a structured diagnostic psychiatric interview for DSM-IV and ICD-10. *Journal of Clinical Psychiatry, 59(S20)*, 22-33.

24. National Health and Medical Research Council (NHMRC) (2007). *National Statement on Ethical Conduct in Human Research*. Canberra, NHMRC.

11. APPENDICES

11.1 QUESTIONNAIRES

Questionnaires to be included in the project are:

PHQ-9: Patient Health Questionnaire – Nine item, (Kroenke, Spitzer, & Williams, 2001)

GAD-7: Generalized Anxiety Disorder-7 Item Scale (Spitzer, Kroenke, Williams, & Löwe, 2006)

DASS-21: Depression, Anxiety and Stress Scales-21 Item, (Lovibond & Lovibond, 1995)

PDSS-SR: The Panic Disorder Severity Rating Scale-Self Report (Houck, Spiegel, Shear, Rucci, 2002)

SP-12: The Social Phobia-12 (Peters, Sunderland, Andrews, Rapee & Mattick, submitted for publication)

WHODAS-II: World Health Organisation Disability Assessment Schedule – 2nd Edition (2001)

K-10: K-10: Kessler – 10 Item (Kessler, Andrews, Colpe et al, 2002)

NEO-FF-I, N scale: NEO- Five Factor Inventory, Neuroticism scale (Costa & McRae, 1985)
